# Exploring the Effects of Cyclosporin A to Isocyclosporin A Rearrangement on Ion Mobility Separation

**DOI:** 10.1021/acs.analchem.3c05165

**Published:** 2024-03-02

**Authors:** Hynek Mácha, Jakub Zápal, Marek Kuzma, Dominika Luptáková, Karel Lemr, Vladimír Havlíček

**Affiliations:** † Institute of Microbiology of the Czech Academy of Sciences, Vídeňská 1083, Prague 142 00, Czech Republic; ‡ Department of Analytical Chemistry, Faculty of Science, Palacký University, 17. listopadu 12, Olomouc 771 46, Czech Republic

## Abstract

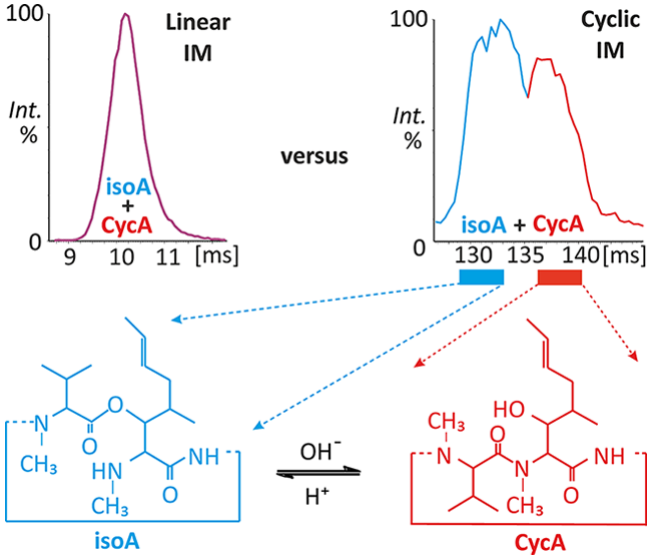

Cyclosporin A (CycA) is a peptide secondary metabolite derived from fungi that plays a crucial role in transplantation surgery. Cyclic traveling wave ion mobility mass spectrometry (IM-MS) revealed an N → O peptidyl shift in singly protonated CycA to isocyclosporin A (isoA), whereas no such isomerization was observed for doubly protonated and sodiated molecules. CycA and isoA were able to be separated by considering doubly protonated precursors using a specific ion fragment. In parallel, sodium ion stabilization facilitated the simultaneous separation and quantitation of singly charged cyclosporin isomers with the limit of detection and coefficient of determination of 1.3% and 0.9908 for CycA in isoA and 1.0% and 0.9830 for isoA in CycA, respectively. Finally, ^1^H–^13^C gHSQC NMR experiments permitted parallel recording of up to 11 cyclosporin conformers. The ratios were determined by integrating the volume of cross-peaks of the upfield resonating hydrogen in the diastereotopic methylene group of sarcosine-3.

Cyclosporin A (CycA), originally isolated from *Trichoderma polysporum*,^1^ is a hydrophobic cyclic undecapeptide with amino acid sequence cyclo[MeBmt(2-butenyl-4,*N*-dimethylthreonine)^1^-Abu^2^-Sar^3^-l-MeLeu^4^-l-Val^5^-l-MeLeu^6^-l-Ala^7^-d-Ala^8^-l-MeLeu^9^-l-MeLeu^10^-l-MeVal^11^]. It has primarily been used as an immunosuppressant, although it also displays antifungal, antiparasitic, and anti-inflammatory properties.^2^ CycA is a prototype compound for pharmaceutical development, and different analogs have been synthesized.^3^ Although it violates Lipinski’s rule of five,^4^ which considers drug solubility and permeability, it demonstrates superb bioavailability due to its conformational variability.^5,6^ Besides solution pH, its conformations are affected by the solvent composition^7,8^ and metal complexation.^9−11^ In protic systems, more cyclosporin conformers are formed than in aprotic solvents.^7,8,12,13^

In the liquid phase, CycA undergoes N → O acid-catalyzed peptidyl rearrangement to form isocyclosporin A (isoA) initiated by the β-hydroxyl group present in the side chain of amino acid MeBmt.^14^ In protic solutions, isomerization occurs over several hours,^15^ and contributes significantly to the degradation of CycA.^16^ CycA rearrangement has also been observed in the gas phase, specifically on a millisecond time scale when analyzing singly protonated molecules of CycA with an ion trap or quadrupole mass analyzers.^17^ The N → O peptidyl shift has been shown to be suppressed in B/E sector and time-of-flight (TOF) instruments operating at a microsecond time frame.^17^ Since rearrangement in singly protonated species can make it difficult to distinguish CycA from isoA, collision-induced dissociation (CID) of doubly protonated species^18^ and metal complexation have been used to differentiate these isomers.^19^ Remarkably, the discussed rearrangement has not been mentioned in any previous ion mobility-mass spectrometry (IM-MS) study of CycA.^20−23^

In IM-MS, intramolecular folding forces dictate different gas-phase packing efficiencies, and the solvent selection can influence the number of gas-phase conformations.^24−26^ Use of a matrix-assisted laser desorption/ionization (MALDI)-IM-TOF MS instrument equipped with an IM drift cell yielded collision cross-section (CCS) values of 296.6 ± 1.2 and 289.9 ± 1.2 Å^2^ for CycA [M + H]^+^ and [M + Na]^+^ ions, respectively.^20^ Regarding [M + H]^+^ ions, a smaller CCS value of sodiated CycA molecules may indicate additional shrinking of the molecular structure caused by the sodium cation, as supported by modeling molecular dynamics.^20^ Drift tube IM-MS measurements revealed two populations of singly protonated CycA molecules centered at CCS values of 271 ± 5 and 282 ± 5 Å^2^, indicating the presence of a conformational ensemble.^22^ In a differential mobility spectrometry (DMS) study, singly protonated CycA and isoA isomers were not separated showing the same compensation voltage shift (∼+6.0 V), but their doubly protonated molecules were well resolved.^21^

The current literature is not consistent with regard to the IM behavior of doubly protonated cyclosporin molecules. Two conformers of [M + 2H]^2+^ ions in CycA with calibrated CCS values of 266 and 275 Å^2^ were observed using nano electrospray ionization (nano-ESI) traveling-wave ion-mobility spectrometry–mass spectrometry (TWIMS-MS).^23^ In DMS, the doubly protonated CycA precursor also exhibited a doublet in the mobilogram.^21^ On the other hand, Do and co-workers reported only one major population of doubly protonated CycA, with CCS of 297 Å^2^.^22^

The present study investigated the impact of the N → O peptidyl shift on the IM separation of CycA and isoA by isolating [M + H]^+^, [M + 2H]^2+^, and [M + Na]^+^ species in the gas phase. NMR spectroscopy was used as a comparative tool for analyzing the isomeric purity and conformational changes in the liquid phase.

## Experimental Section

### Chemicals

Samples of CycA and isoA were provided by TEVA Czech Industries (Opava-Komárov, Czech Republic). HPLC grade methanol, trifluoroacetic acid (TFA), formic acid (FA), acetonitrile (ACN), and LC-MS grade water were purchased from Honeywell (Prague, Czech Republic). Deuterated solvents CD_2_Cl_2_ (99.80%D), CD_3_OD (99.80%D), and D_2_O (99.96%D) were purchased from VWR (Prague, Czech Republic), sodium trifluoroacetate (NaTFA), and α-cyano-4-hydroxycinnamic acid (CHCA) were from Sigma-Aldrich (Prague, Czech Republic), sodium iodide (NaI) was from Waters Corporation (Wilmslow, U.K.) and peptide calibration standard II was from Bruker Daltonics (Bremen, Germany).

### Mass Spectrometry Fragmentation Experiments

In MALDI analyses, cyclosporins (4.15 μM, 50% aqueous methanol) were individually spotted (1 μL) on a target plate and overlaid with a CHCA matrix (10 mg/mL, 50% ACN/0.1% TFA, 1 μL). Ultraflex III MALDI-TOF/TOF and solariX 12T Fourier-transform ion cyclotron resonance (FTICR) mass spectrometers (both Bruker Daltonics, Billerica, U.S.A.) operated in positive-ion mode were calibrated using peptide calibration standard II in the mass range 40–2220 *m*/*z*. Product ion mass spectra of [M + H]^+^ precursors were acquired using Smartbeam (21% power, LIFT technology) and Smartbeam II (30% power, CID in a quadrupole) lasers, respectively.

For ESI in the solariX 12T FTICR spectrometer was operated in positive-ion mode; data were acquired in the mass range 100–1300 *m*/*z*, with an accumulation time of 0.1 s, and external calibration to ion clusters of NaTFA. Data were collected with 4 M data points in the transient providing an estimated resolving power of 53000 at *m*/*z* 400. The ESI parameters were optimized regarding the absolute ion intensity with a capillary voltage of 4.6 kV, nebulizer gas pressure of 0.3 bar, and TOF of 1.2 ms. Cyclosporins were dissolved in methanol (0.17 μM), and analyzed in less than 1 h. [M + H]^+^ ions were fragmented at a collision voltage of 20 V. For naturally generated [M + Na]^+^ ions (no sodium added), the collision voltages were 50 and 35 V for CycA and isoA, respectively. Data were processed using DataAnalysis 5.0 and CycloBranch^27^ software.

### Ion Mobility Spectrometry

IM experiments were carried out using linear TWIMS Q-TOF (SYNAPT G2-Si, Waters, U.K.) and cyclic TWIMS Q-TOF (SELECT SERIES Cyclic IMS, Waters, U.K.) spectrometers operated in positive ion mode. Both instruments were calibrated using NaI in the mass range 100–2000 *m*/*z*. Cyclosporins (0.166 or 0.832 μM) were dissolved in 50% aqueous or 100% methanol, and the fresh solutions were directly infused to the ESI source. The following settings were used for linear TWIMS: spray voltage 3 kV, source offset 50 V, cone voltage 150 V, helium flow rate 200 mL/min, nitrogen flow rate 110 mL/min, wave amplitude 40 V, and wave velocity 400 m/s. CID experiments were performed in a postmobility transfer cell with collision voltages of 98 and 59 V for [M + Na]^+^ and [M + H]^+^ ions, respectively.

Cyclic TWIMS was performed with capillary voltages of 3, 4, and 2 kV for singly protonated, doubly protonated, and sodiated molecules, respectively. The ion source was held at an offset voltage of 10 V and cone voltage of 40 V. Five (sodiated ions), four (doubly protonated ions), and three (singly protonated ions) passes through a mobility cyclic cell were carried out with helium flow rate 120 mL/min, nitrogen flow rate 40 mL/min, a wave amplitude of 18 V and a wave velocity of 375 m/s. CID experiments were performed using a postmobility transfer cell and collision voltages of 60, 23, and 105 V for singly protonated, doubly protonated, and sodiated molecules, respectively.

Protonated molecules (*m*/*z* 1202.8) were generated through electrospraying a 0.832 μM CycA solution in methanol: aqueous 5 mM NH_4_Ac (1:1, v/v). The [M + H]^+^ ions selected in a quadrupole underwent three passes in the mobility cell before ion slices being isolated at variable drift times (140, 144, 150, and 157 ms with a 2 ms window each) and sent to a prestore. The reinjection into the mobility cell was performed with two collisional activations (0 and 60 V). Each slice then was separated by three passes.

For quantitation, CycA and isoA standards were mixed (CycA content: 0%, 5%, 10%, 25%, 50%, 75%, 90%, 95%, 98%, 99%, or 100%, total concentration of both cyclosporins = 1000 ng/mL) and measured in six technical replicates. IM-MS data were evaluated using MassLynx 4.2 software (Waters, U.K.). Outliers were excluded, and calibration curves were constructed using OriginPro 2022 software (OriginLab, Northampton, U.S.A.). Calibration curves were obtained by a linear regression analysis. The limit of detection (LOD) and quantitation (LOQ) were estimated from the standard deviation of the *y*-intercept of the regression line from the three lowest edge points (LOD = 3.3 × SD_int_/slope; LOQ = 10 × SD_int_/slope).

### NMR Spectroscopy

NMR spectra were acquired at 293.2 K in CD_2_Cl_2_, CD_3_OD or a mixture of CD_3_OD and D_2_O (1:1, v/v) using Bruker AVANCE III 600 and 700 MHz NMR spectrometers (Bruker Biospin GmbH, Germany) controlled with Topspin 3.6 software (Bruker Biospin GmbH, Germany). Chemical shifts were calibrated using dichloromethane (δ_H_ = 5.323 ppm, δ_C_ = 53.87 ppm) and methanol (δ_H_ = 3.398 ppm, δ_C_ = 48.20 ppm, 100% or 50% aqueous CD_3_OD) as an internal standard.

For sample purity determination, ^1^H NMR and ^1^H–^13^C gHSQC (heteronuclear single quantum correlation) spectra in CD_2_Cl_2_ were compared to our previously collected data for a complete set of experiments (^1^H NMR, ^13^C NMR, gCOSY, ^1^H–^13^C gHSQC, ^1^H–^13^C gHMBC, *J*-resolved, ROESY, ^1^H–^13^C gHSQC-TOCSY, ^1^H–^15^N gHSQC and ^1^H–^15^N HMBC). The relative molar CycA and isoA ratios were determined by integration of −NH– signals in the ^1^H NMR spectrum at similar concentrations since the corresponding chemical shifts may have been concentration-dependent.

^1^H–^13^C gHSQC NMR experiments were conducted using a mixture of CD_3_OD/D_2_O (50:50, v/v) or 100% CD_3_OD to determine the relative molar ratio of the distinct spatial conformers of CycA and isoA in solution. The ratios were determined by integrating the volume of cross-peaks of the upfield resonating hydrogen in the diastereotopic methylene group of sarcosine (Sar-3).

## Results and Discussion

### Cyclosporin A and Isocyclosporin A Purity and Stability in CD_2_Cl_2_

The supplier-declared purities, determined by HPLC-UV, were 99.4% and 92.9% for CycA and isoA, respectively. Furthermore, ^1^H NMR data of CycA analysis in aprotic CD_2_Cl_2_ demonstrated the long-term high stability needed for 2D NMR experiments (Supporting Information, Figure S1). The ^1^H–^13^C gHSQC spectrum of CycA confirmed no isoA contamination (Supporting Information, Figure S2). The ROESY spectrum of CycA (data not shown) revealed chemical exchange between the minor signal sets and the main set of CycA signals, indicating that the minor sets belong to CycA conformers (given the time scale of CycA to isoA isomerization in CD_2_Cl_2_).

In contrast, NMR spectroscopy detected some CycA signals in the fresh solution of the isoA standard (Supporting Information, Figures S3 and S4). The overlay of α-methine signals in proton-edited ^1^H–^13^C gHSQC spectra indicated that the isoA standard contained 9% CycA (Supporting Information, Figure S5). Complete NMR data for the dominant isoA conformer in CD_2_Cl_2_ are provided in Supporting Information, Table S1.

### Cyclic TWIMS Revealed an N → O Peptidyl Shift in Singly Protonated Cyclosporin A

**Figure 1:**
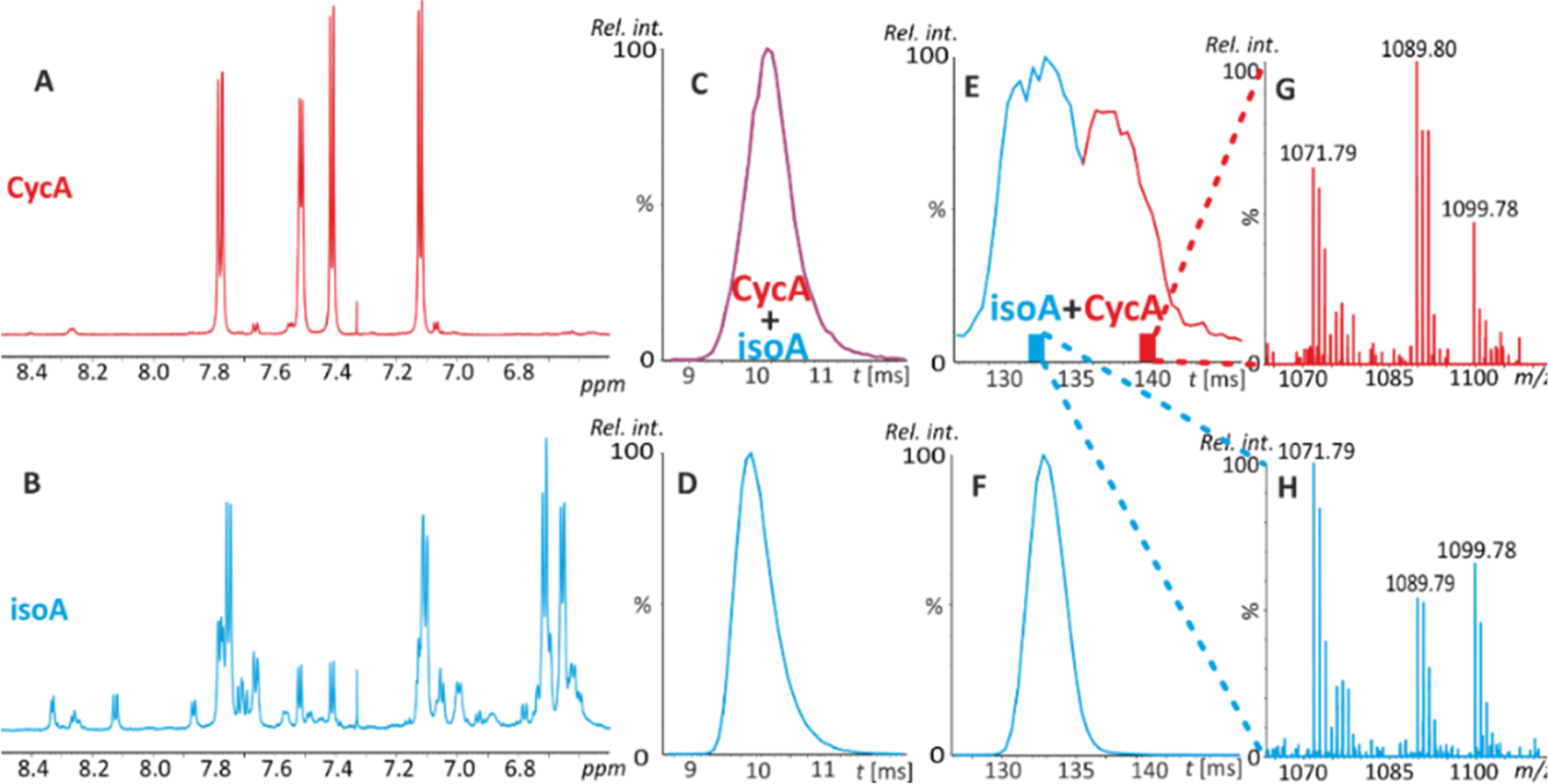
Distinguishing CycA from isoA in the liquid and gas phase. The diagnostic −NH– region of the ^1^H NMR spectra of CycA (A) and isoA (B) standards are colored red and blue, respectively. Singly protonated molecules of both compounds were analyzed using linear TWIMS (C, D) and cyclic TWIMS with thee passes (E, F). Low content of CycA in 1F is not detected due to partly conversion and low protonation efficiency of CycA. Additionally, postcyclic mobility CID spectra of the *m*/*z* 1202.8 precursor with a collision voltage of 60 V were collected and are shown for 133 and 140 ms drift times (G, H). The mass spectrometry experiments were conducted in 100% methanol.

While distinguishing between CycA and isoA in the liquid state appeared facile (Figure 1A,B), the N → O peptidyl rearrangement in [M + H]^+^ ions of CycA rendered the gas-phase analysis complicated. In linear TWIMS signals, the peak maxima of isomers were slightly shifted due to the strong overlap of the protonated molecules (Figure 1C,D).

Cyclic TWIMS of CycA showed a wider doublet with the first maximum corresponding to the isoA mobility peak, which was narrow and symmetric (compare Figure 1E,F). Owing to rearrangement,^17^ CycA can partly convert to isoA during analysis. Therefore, its mobilogram may correspond to an isomeric mixture. The effect of this rearrangement was confirmed by comparing fragmentation spectral patterns corresponding to drift times of approximately 133 and 140 ms, which are specific to isoA and CycA, respectively (Figure 1G,H).

**Figure 2:**
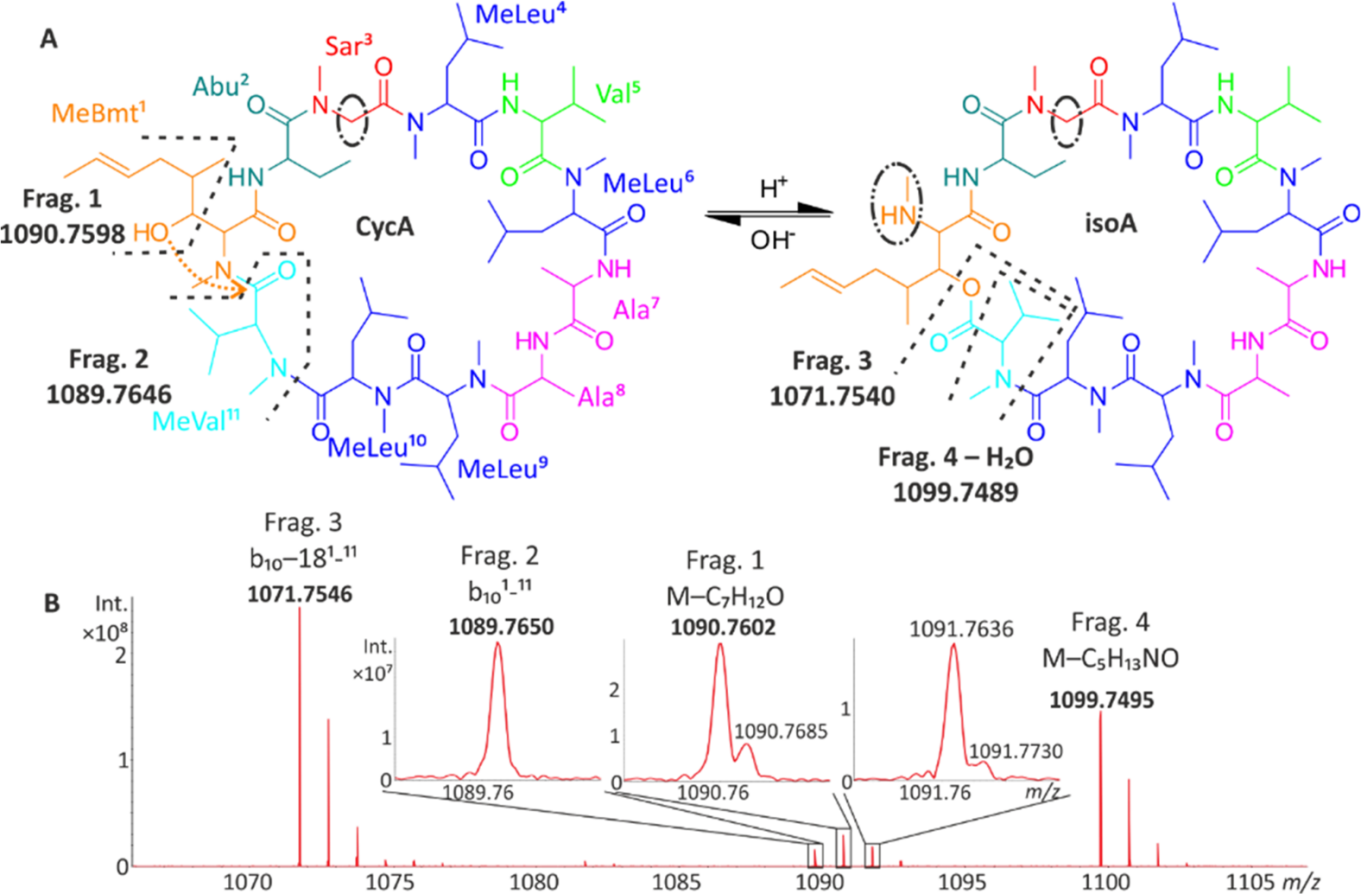
Cyclosporin A behavior in the gas phase. Reversible isomerization of CycA to isoA (A) due to N → O acyl migration via a hydroxyoxazolidine intermediate.^14^ The suggested diagnostic ion structures follow the peptide nomenclature,^28,29^ where the superscript indicates the amino acid pair between which the primary ring opening occurs. Product ion mass spectrum of the protonated CycA molecule recorded on an ESI-FTICR mass spectrometer in 100% methanol (B).

The spectrum at 133 ms matched the fragmentation spectrum of singly protonated isoA acquired by cyclic TWIMS (data not shown). Furthermore, analyzing the mass-to-charge ratio profiles of three diagnostic ions (*m*/*z* 1071.78, 1089.80, 1099.78) recorded for the CycA standard provided convincing evidence that isoA was present (Supporting Information, Figure S6). The dominant signals of isoA fragmentation, i.e., at *m*/*z* 1071.78 and 1099.78, corresponded to the first peak of a doublet, whereas the signal for the ion at *m*/*z* 1089.80 was uniformly distributed. The proposed diagnostic ion structures are shown in Figure 2. The peak at *m*/*z* 1090.76 interfered with the 1089.76 isotopic cluster and corresponded to a MeBmt side chain loss.

Within the isoA structure, primary protonation may occur at the nitrogen atom of the methylamine group. Methylamine elimination from the protonated molecule yielded an ion at *m*/*z* 1171.8064 (spectrum not shown). It is important to consider that the low content (approximately 9%) of CycA detected in the isoA standard by NMR may have slightly affected the fragmentation pattern.

Increasing the number of passes through the cyclic mobility cell from 1 to 3 did not significantly affect the intensity ratio (1.46 versus 1.53) of the diagnostic ions at *m*/*z* 1089 and 1099 (Supporting Information, Figure S7). This indicates that an isomeric equilibrium had been achieved prior to the ion cloud entering the cyclic mobility cell. The additional slicing experiment confirmed this statement. In the experiment, singly protonated CycA molecules underwent three passes in the cyclic mobility cell. 2 ms ion slices at variable drift times were then sent to a prestore and reinjected back to the mobility cell for an additional three passes. The narrow peak shapes for each 2 ms slice (Supporting Information, Figure S8) confirm that the interconversion of CycA to isoA must occur during the ionization process or in ion optics before mobility separation. Finally, CycA to isoA isomerization within a mass spectrometer occurred using both ESI and MALDI (Supporting Information, Figure S9).

### Separation of CycA from isoA in [M + 2H]^2+^ Ions was Enabled through an *m*/*z* 212 Fragment Ion

**Figure 3:**
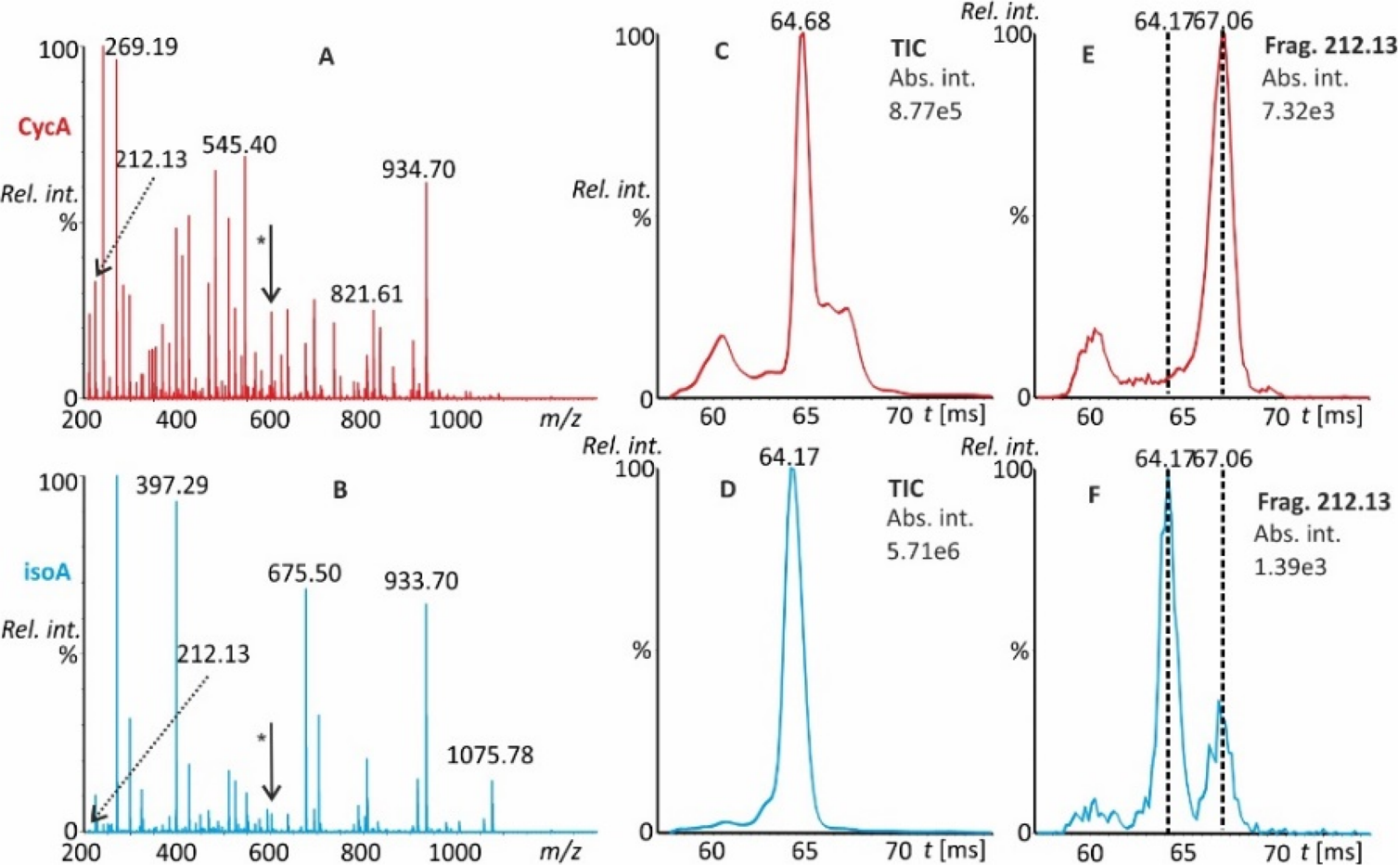
Fragmentation spectra of doubly protonated molecules (*[M + 2H]^2+^ = 601.95 *m*/*z*) of CycA (A) and isoA (B) after four passes through a TWIMS in a transfer cell held at a collision voltage of 23 V. The total ion current ATD of CycA (C) and isoA (D) shows the comparable mobilities of the dominant conformers. The distinguishing of CycA (E) from isoA (F) can be achieved by considering the *m*/*z* 212.13 fragment.

The signal for [M + 2H]^2+^ ions almost overlapped in a cyclic TWIMS experiment (Figure 3). Interestingly, distinguishing between CycA and isoA could be achieved using the ATD profiles of fragment ion *m*/*z* 212.13 (Figure 3E,F). For CycA, this fragment appears at 67.06 ms. Precursor characteristic drift times of 67.06 and 64.17 ms were recorded for CycA (minor conformer) and isoA, respectively. The absence of a signal for *m*/*z* 212 at 64.17 ms in the mobilogram of CycA indicated the absence of isoA in the highly pure CycA sample. Conversely, the appearance of a signal at *m*/*z* 212 with a 67.06 ms drift time, specific for CycA, confirmed CycA contamination in the isoA solution (Figure 3E, F). Note the NMR-spectroscopy-deduced CycA content in isoA (approximately 9%). No N → O peptidyl shift occurred in the [M + 2H]^2+^ ions of CycA, as previously reported.^18^

### Stabilization by Sodium Ion Enabled the Mutual Quantitation of Cyclosporin Isomers

**Figure 4:**
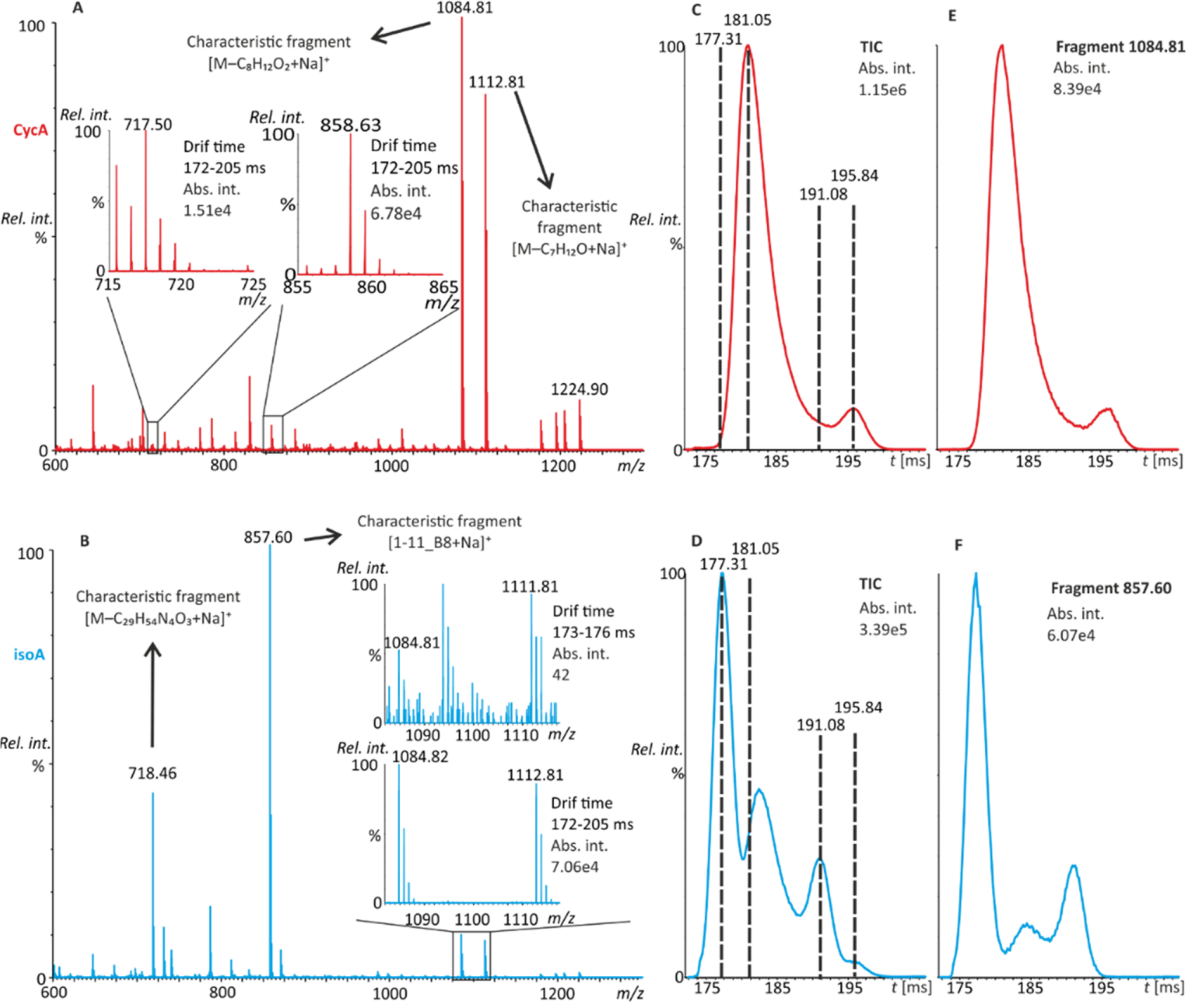
Cyclic TWIMS fragmentation spectra of singly sodiated molecules of CycA (A) and isoA (B). The insets indicate the possible presence and absence of the interfering cyclosporin isomers. Additionally, the total ion current ATD of [M + Na]^+^ ions of CycA (C) and isoA (D), along with the extracted ion mobilograms of their dominant fragments (E, F) after five passes in the mobility cell, are presented. All data were collected in 50% aqueous methanol.

CycA and isoA have high but different affinities for sodium cations.^19^ In the ESI-Q-FTICR fragmentation of CycA sodiated molecules (naturally generated [M + Na]^+^ ions with no extra sodium added), two characteristic fragment ions were observed. The first, at *m*/*z* 1112.7413 (1112.7418 calculated for C_55_H_99_N_11_O_11_Na), a sodiated variant of *m*/*z* 1090.7602 (Figure 2), resulted from the elimination of C_7_H_12_O and was attributed to side chain loss in MeBmt (Figure 4A). The second ion, at *m*/*z* 1084.7464 (1084.7469 calculated for C_54_H_99_N_11_O_10_Na), was attributed to the elimination of C_8_H_12_O_2_ from the sodiated molecule, side chain loss and the additional loss of carbon monoxide.^30^ The spectrum of the isoA sodium adduct was dominated by a b_8_ ion at *m*/*z* 857.5467 (calculated at 857.5471 for C_42_H_74_N_8_O_9_Na), which corresponded to ring opening between the first and 11th amino acid. Additionally, a second abundant fragment ion at *m*/*z* 718.4107 (calculated at 718.4110 for C_33_H_58_N_7_O_9_Na) emerged from the loss of C_29_H_54_N_4_O_3_ (Figure 4B). The nature of the product ions was inferred from the accurate product ion mass spectra of CycA and isoA recorded on a Q-ESI-FTICR mass spectrometer in 50% aqueous methanol (Supporting Information, Figure S10).

In cyclic TWIMS experiments, the ATD profiles of CycA/isoA sodiated molecules (*m*/*z* 1224.90) revealed marked differences in the total ion current (TIC) mobilograms (Figure 4C,D) and cyclosporin-characteristic fragment ions (Figure 4E,F). In accordance with the NMR spectroscopy data, a minor contribution of CycA to the signal of the TIC at *m*/*z* 1084 was present in isoA (Figure 4B, inset). Conversely, the characteristic isoA signal at *m*/*z* 857.60 was not detected in the CycA sample (Figure 4A, inset).

The IM separation combined with the high detection sensitivity of sodiated molecules enabled the quantification of both cyclosporin analogues in the mixtures. Calibration curves were constructed using the characteristic fragment ion mobility peaks (*m*/*z* 1084.81 for CycA and *m*/*z* 857.60 for isoA) with coefficients of determination of 0.9908 and 0.9830, respectively. The LOD and LOQ for CycA content in isoA were 1.3% and 4.0% (total amount of 1000 ng/mL), respectively, representing 13 and 40 ng/mL. The LOD and LOQ for isoA content in CycA were 1.0% and 3.0% (10 and 30 ng/mL), respectively (Supporting Information, Figure S11). The ATD profiles of individual isomers in the gas phase (their sodiated molecules) were not influenced by the N → O peptidyl rearrangement.

### Cyclosporins Showed Higher Conformational Flexibility in the Protic Liquid Phase than in the Gas Phase

**Figure 5:**
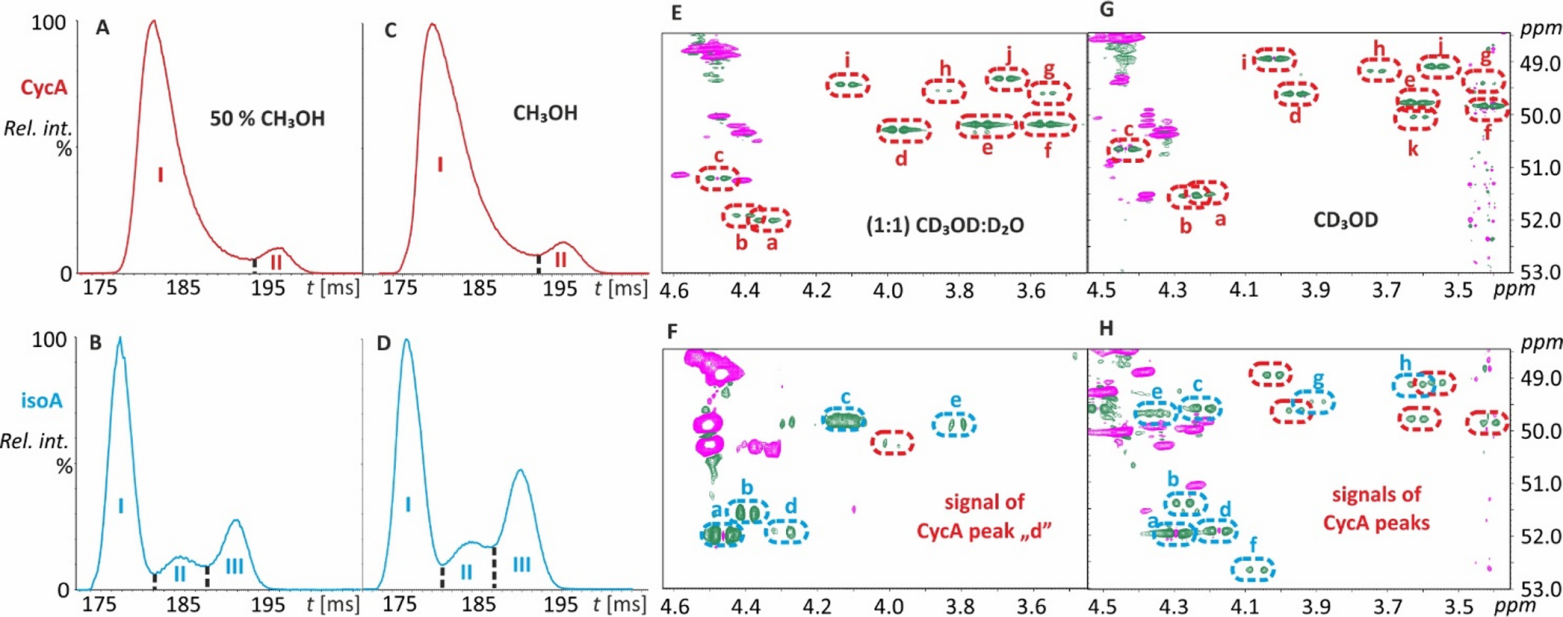
Characteristic fragment ion (CycA *m*/*z* 1084.81, isoA *m*/*z* 857.60) mobilograms recorded in 50% (A, B) and 100% methanol (C, D). 2D ^1^H–^13^C HSQC NMR spectra of cyclosporin isomers zoomed into the region of upfield signals of the diastereotopic −CH_2_– group in the Sar-3 building block (dashed ovals) recorded in (1:1) CD_3_OD:D_2_O (E, F) and CD_3_OD (G, H). The signals of CycA in isoA are consistent with the original level of impurity demonstrated in the CD_2_Cl_2_ experiment. I–III and a–f conformers were collected by IM-MS and NMR spectroscopy, respectively.

To explore the CycA/isoA conformational space in protic solvents, solution NMR data were compared with data obtained by IM-MS. In the IM-MS gas phase separation of sodiated molecules, the solvent composition (aqueous 50% versus 100% methanol) had little effect. Spraying CycA and isoA solutions showed at least two and three peaks (denoted as I, II, and III forms), respectively (Figure 5A–D). A minor difference was noted in isoA (additional form III), which did not compromise CycA/isoA mutual discrimination or separation.

Based on NMR spectroscopy, the conformational space was probed using a distinctive Sar-3 building block primarily via the ^1^H–^13^C gHSQC methylene signals, differentiated by the altered spatial arrangement in its vicinity. CycA and isoA in D_2_O/CD_3_OD (50/50, v/v) were resolved into at least 10 (a–j) and 5 (a–e) detectable signals, respectively (Figure 5E,F), each representing an individual conformer. In pure CD_3_OD, the situation was even more complicated, as CycA and isoA showed at least 11 and 8 individual detectable conformers, respectively (Figure 5G,H). When these two solvent systems were compared, variable relative conformer ratios and drifts in chemical shifts of the respective signals were noticed. These changes were more pronounced in the case of isoA. Consequently, the peaks assignments (a–h) in different solvents may not represent the same conformers, but the data provide the overall relative conformer composition in a sample (Table 1).

**Table 1:** Relative Mobility Peak Area and Relative Molar Ratios of Conformers of Cyclosporin Isomers Recorded by Ion Mobility Spectrometry and NMR Spectroscopy in Protic Solvents

		50% CH_3_OH			CH_3_OH				(1:1) CD_3_OD/D_2_O					CD_3_OD					
CycA	peak	I	II		I	II		peak	a	b	c	d	e	a	b	c	d	e	f
								mol. ratio	5	2	5	27	25	4	4	9	10	17	24
	AUC %^a^	93	7		92	8		peak	f	g	h	i	j	g	h	i	j	k	
								mol. ratio	19	1	1	6	9	1	2	17	12	1	
isoA	peak	I	II	III	I	II	III	peak	a	b	c	d	e	a	b	c	d	e	f
								mol. ratio	43	14	32	6	5	51	7	13	12	10	3
	AUC %^a^	65	13	22	55	13	32	peak						g	h				
								mol. ratio						1	3				

a AUC: area under the curve.

## Conclusions

The rearrangement of CycA to isoA can potentially impede IM and MS analyses by increasing the intensity of isoA signals. In the present study, this was demonstrated during linear and cyclic TWIMS mass spectrometry experiments for singly protonated molecules produced by ESI. The effect not only hampers quantitative analysis but can also distort CCS determination and conformational studies of cyclosporins, e.g., influencing mobility peak maxima due to the isoA signal. Notably, the presence of a β-hydroxyl group may also affect analyses of other cyclosporins or structurally similar peptides.

Sodium adducts were utilized to measure the isomer content in mixtures by using characteristic ATD profiles and MS/MS spectra. Calibration curves exhibited good linearity, with LOD and coefficient of determination of 1.3% and 0.9908 for CycA in isoA, and 1.0% and 0.9830 for isoA in CycA, respectively. Use of sodium adducts for the separation of CycA and isoA mixtures by IM offers suppression of the N → O peptidyl shift and insensitivity to protic solvent composition. Use of cyclic TWIMS enabled the resolution of only two and three sodiated conformers in the gas phase for CycA and isoA, respectively. The method proposed for analyzing sodium adducts is suitable for quantitative monitoring of CycA during drug manufacture and quality control checks. Additionally, the stabilization of peptide structures by sodium adducts could be applied to similar isomer pairs that undergo N → O peptidyl shifts.

We report an analytically significant observation from ^1^H–^13^C gHSQC NMR experiments, which permits parallel detection and differentiation of up to 11 cyclosporin conformers. The ratios were determined by integrating the volume of cross-peaks of the upfield resonating hydrogen in the diastereotopic methylene group of sarcosine-3. Whereas NMR data revealed significant variations in the number and content of conformers in different solvents, their impact on IM separation was negligible.

## Supporting Information Available

The full NMR spectra of CycA and isoA; ATD of CycA diagnostic fragments; Fragmentation of CycA in cyclic TWIMS and in FTICR generated by ESI and MALDI; Fragmentation spectra of sodiated CycA and isoA; Calibration plots (PDF)
