# Supplementary material for: Exploring the Effects of Cyclosporin A to Isocyclosporin A Rearrangement on Ion Mobility Separation
Source: Anal Chem. 2024 Mar 2;96(10):4163–70. doi: 10.1021/acs.analchem.3c05165 (PMC10938282; doi:10.1021/acs.analchem.3c05165)
Supplement: Supplementary file 1 — ac3c05165_si_001.pdf [file ac3c05165_si_001.pdf]

Supporting information

Exploring the effects of cyclosporin A to isocyclosporin A rearrangement on ion mobility separation

Hynek Mácha[1,2], Jakub Zápal[1], Marek Kuzma[1,2], Dominika Luptáková[1], Karel Lemr[1,2], Vladimír Havlíček[1,2]*

[1]Institute of Microbiology of the Czech Academy of Sciences, Vídeňská 1083, Prague 142 00, Czech Republic

[2]Department of Analytical Chemistry, Faculty of Science, Palacký University, 17. listopadu 12, Olomouc 771 46, Czech Republic

*corresponding author

Table of contents:

Figure S1 ^{1}H NMR spectrum of CycA (700.13 MHz for ^{1}H, CD_2Cl_2, 20°C) demonstrating high sample purity and stability, verified by subsequent repeated ^{1}H NMR acquisition over several days.

Figure S2 ^{1}H-^{13}C gHSQC spectrum of CycA (700.13 MHz for ^{1}H, 176.05 MHz for ^{13}C, CD_2Cl_2, 20°C).

Figure S3 ^{1}H NMR spectrum of isoA (700.13 MHz for ^{1}H, CD_2Cl_2, 20°C).

Figure S4 ^{1}H-^{13}C gHSQC spectrum of isoA (700.13 MHz for ^{1}H, 176.05 MHz for ^{13}C, CD_2Cl_2, 20°C). CycA traces were recorded.

Figure S5 Overlay of α-methine segments in proton-edited ^{1}H-^{13}C gHSQC spectra (700.13 MHz for ^{1}H, 176.05 MHz for ^{13}C, CD_2Cl_2, 20°C) of isoA and CycA. Note CycA contamination (CH-red/CH_2-purple) in the sample of isoA (CH-blue/ CH_2-green). According to the relative ratios of -NH- signal intensities in the ^{1}H NMR spectrum, the signals of CycA represent 9% of all cyclosporins and their conformations in the isoA sample in CD_2Cl_2.

Figure S6 ATD of diagnostic fragments in protonated molecules of CycA.

Figure S7 Fragmentation spectrum of singly protonated CycA after one (**A**) and three (**B**) passes in a cyclic TWIMS cell.

Figure S8 Slicing mobility experiment with the protonated CycA molecules.

Figure S9 Fragmentation spectrum of singly protonated CycA generated by electrospray ionization (**A**) and MALDI (**B**) in FTICR mass spectrometer.

Figure S10 High mass resolution fragmentation spectra of sodiated molecules (*m/z* 1224.80) of CycA (**A**) and isoA (**B**) recorded by FTICR mass spectrometry. In the mass spectrum, peaks are displayed with their nominal masses. Exact mass measurements are provided in the table below. SC: side chain of MeBmt, RC: ring cleavage, CO: carbon and oxide.

Figure S11 Calibration curves for CycA in isoA (A) and isoA in CycA (B). The plots were constructed by calculating the average area under the curve (AUC) in mobilograms of their characteristic fragments at *m/z* 1084.81 (CycA) and 857.60 (isoA).

Table S1 The ^{1}H, ^{13}C, and ^{15}N NMR data of the main conformational isomer of isoA (TEVA standard). Data were collected on NMR spectrometer - Bruker Avance III 600 MHz (600.23 MHz for ^{1}H, 150.94 MHz for ^{13}C, 60.82 MHz for ^{15}N, CD_2Cl_2, 293.2 K).

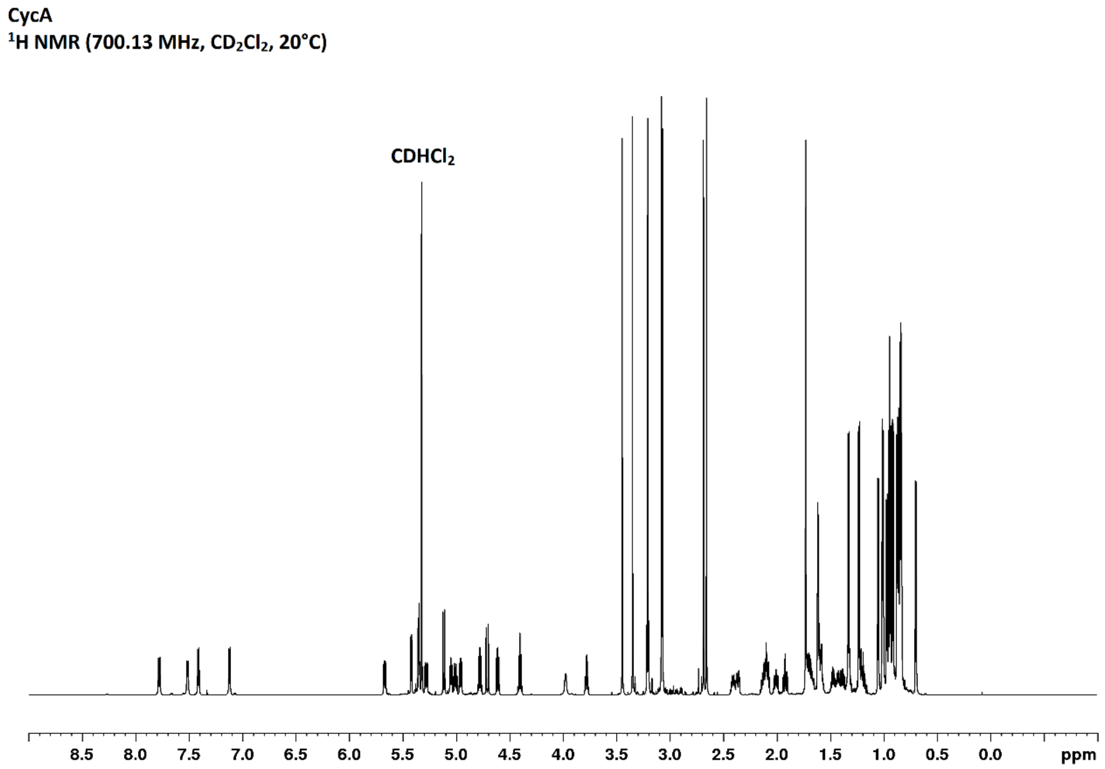

Figure S1 The ^{1}H NMR spectrum of CycA (700.13 MHz for ^{1}H, CD_2Cl_2, 20°C). The high sample purity and stability were revealed and verified by subsequent repeated ^{1}H NMR acquisition over several days.

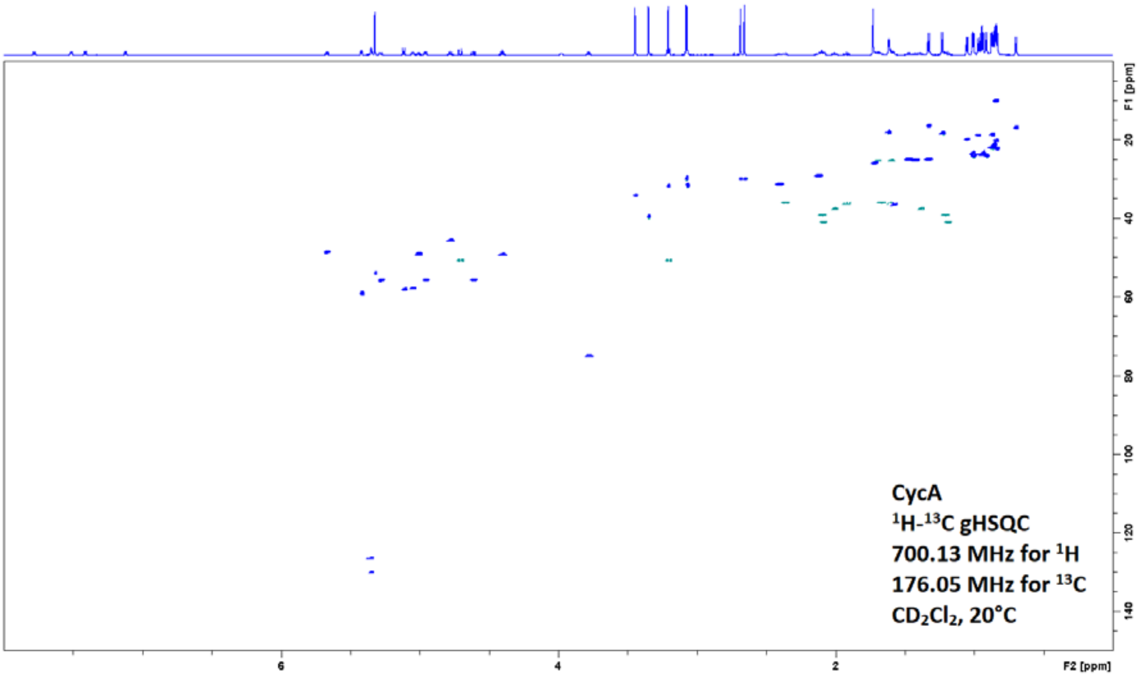

Figure S2 The ^{1}H-^{13}C gHSQC spectrum of CycA (700.13 MHz for ^{1}H, 176.05 MHz for ^{13}C, CD_2Cl_2, 20°C).

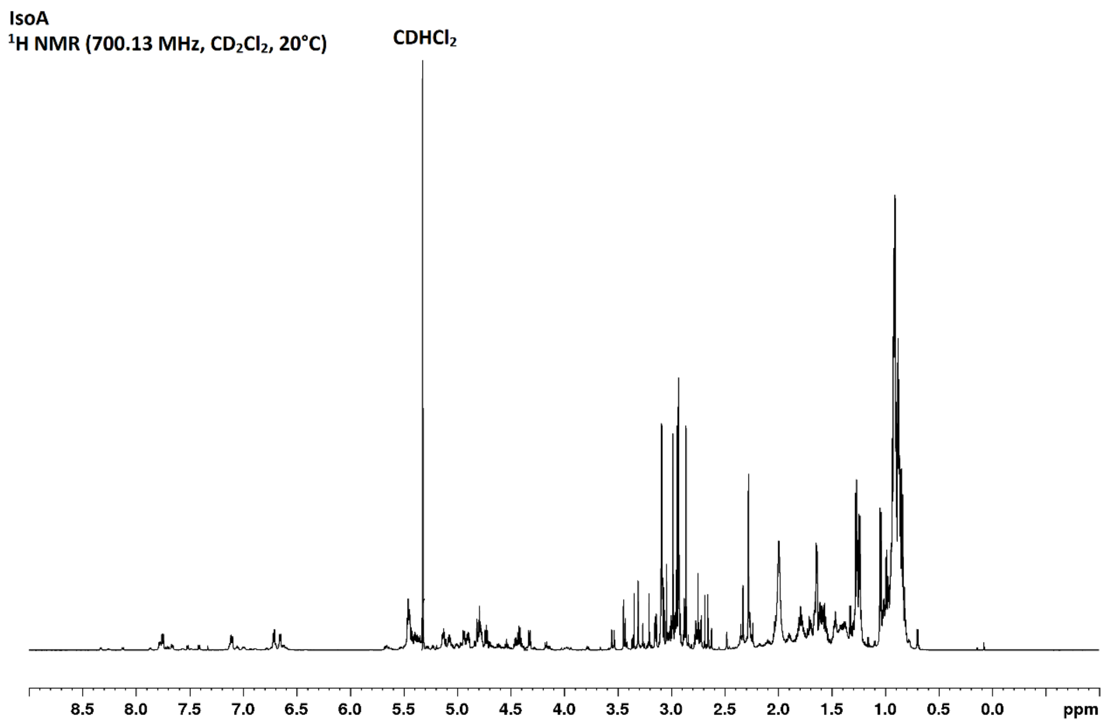

Figure S3 The ^{1}H NMR spectrum of isoA (700.13 MHz for ^{1}H, CD_2Cl_2, 20°C).

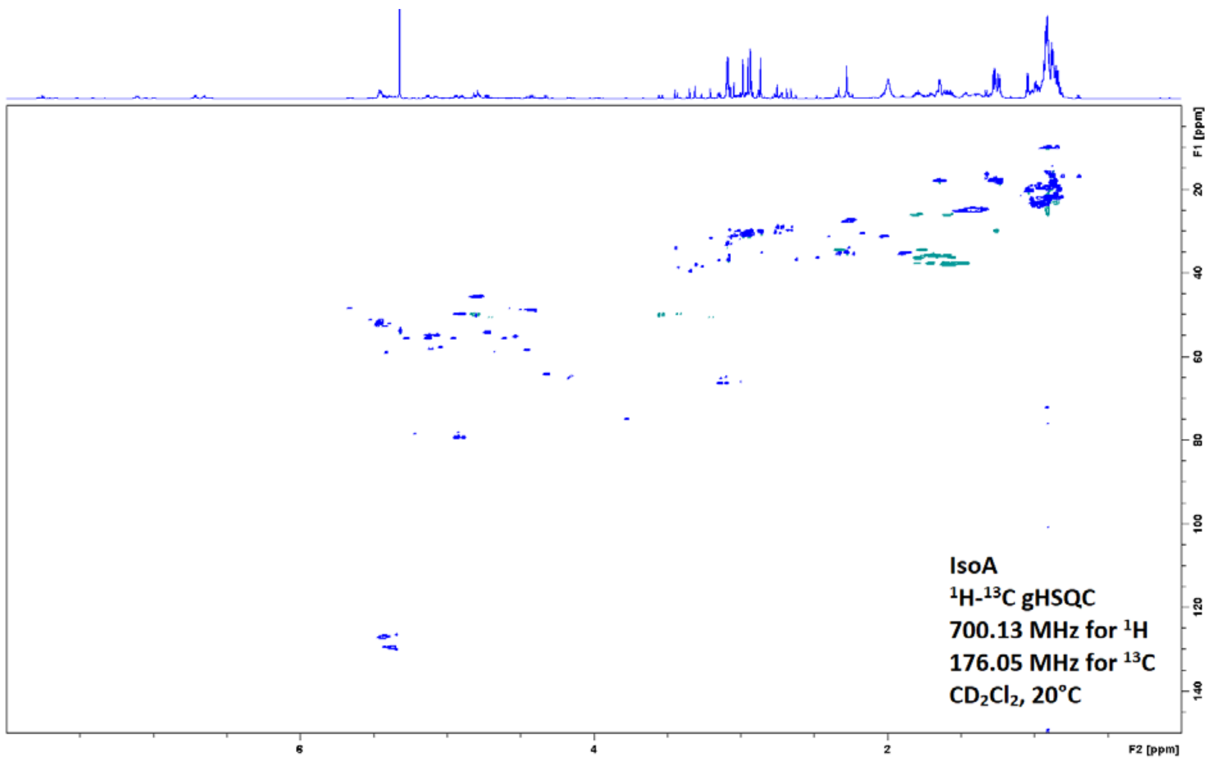

Figure S4 The ^{1}H-^{13}C gHSQC spectrum of isoA (700.13 MHz for ^{1}H, 176.05 MHz for ^{13}C, CD_2Cl_2, 20°C). CycA traces were recorded.

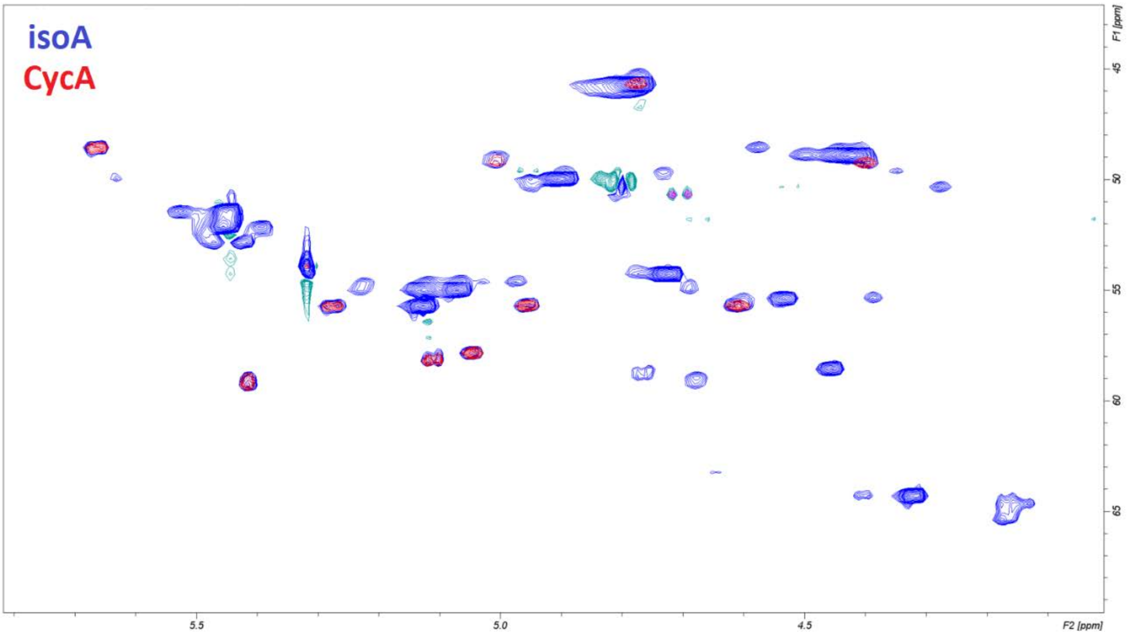

Figure S5 The overlay of α-methine segments in proton-edited ^{1}H-^{13}C gHSQC spectra (700.13 MHz for ^{1}H, 176.05 MHz for ^{13}C, CD_2Cl_2, 20°C) of isoA and CycA. Note CycA contamination (CH-red/CH_2-purple) in the isoA sample (CH-blue/ CH_2-green). According to the relative ratios of -NH- signals intensities in the ^{1}H NMR spectrum, the signals of CycA represent 9 % of all the cyclosporins and their conformations in the isoA sample in the CD_2Cl_2 solution.

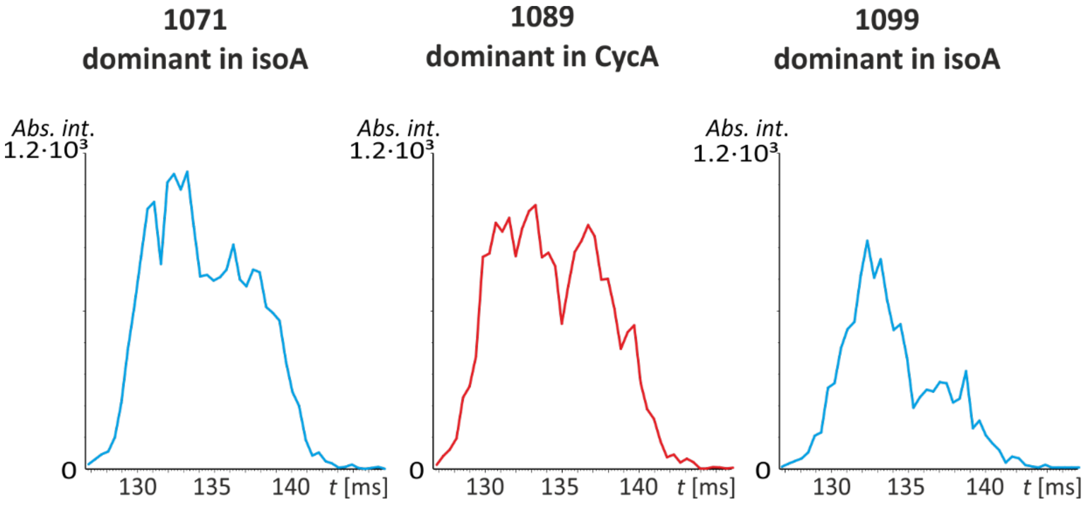

Figure S6 ATD of diagnostic fragments in protonated molecules of CycA.

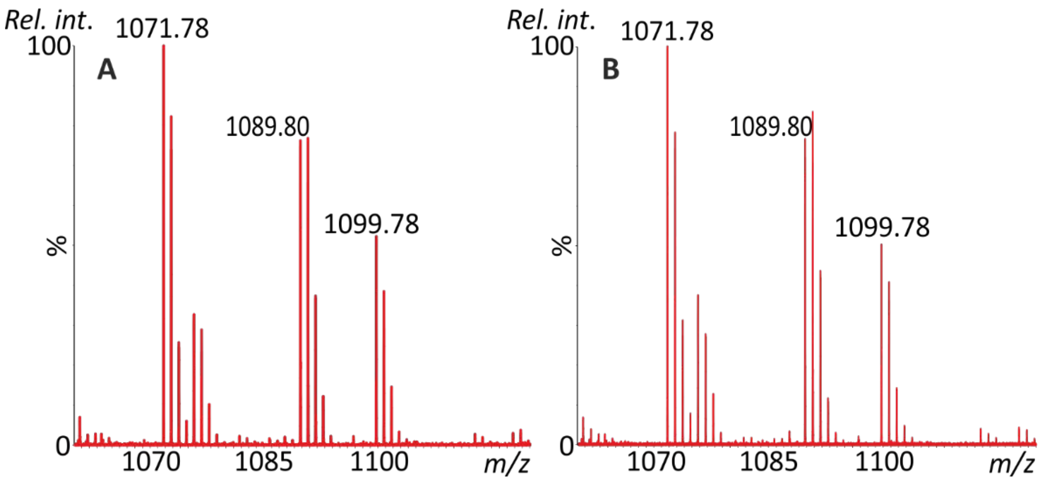

Figure S7 Fragmentation spectrum of singly protonated CycA after one (**A**) and three (**B**) passes in cyclic TWIMS cell.

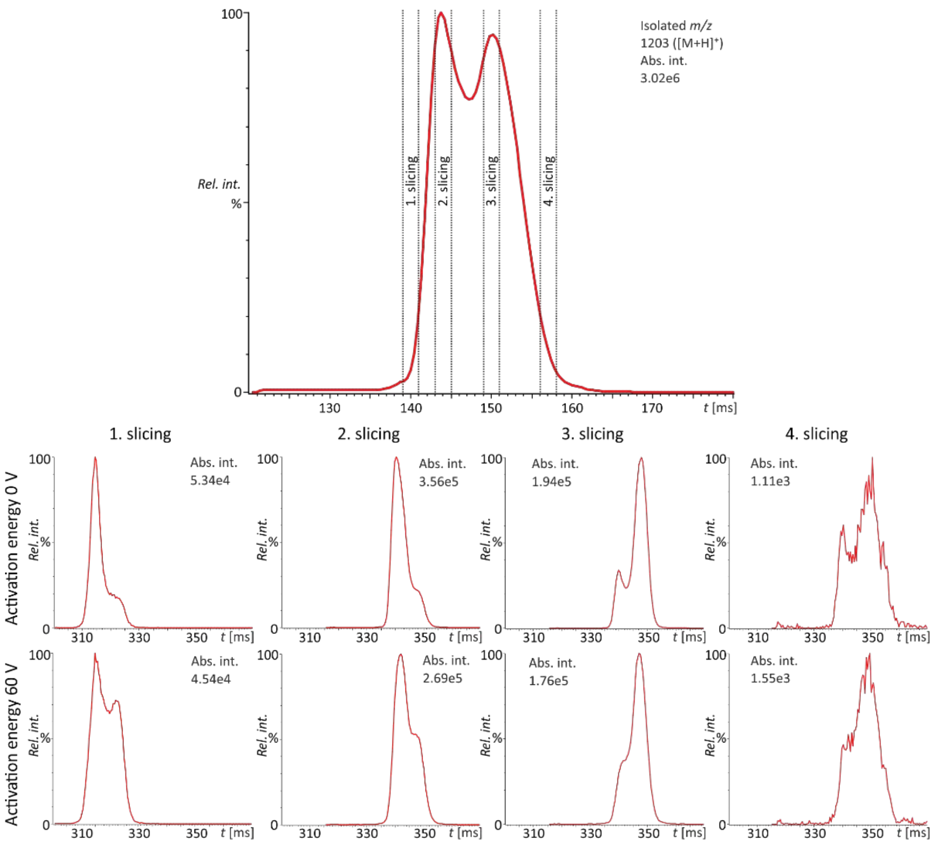

Figure S8: Slicing mobility experiment with the protonated CycA molecules. After their selection in a quadrupole and three passes in the mobility cell, ion slices at the variable times (140, 144, 150, and 157 ms with 2 ms window each) were isolated and sent to a prestore. Their reinjection into the mobility cell was then performed with different collisional activation at 0 V and 60 V, respectively.

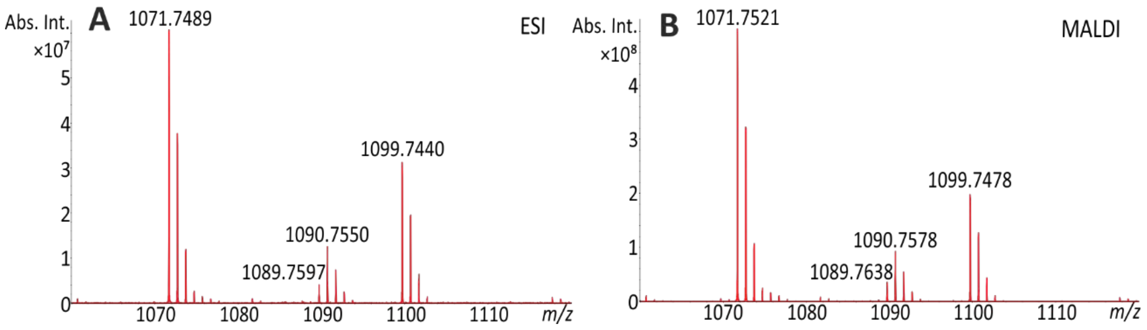

Figure S9 Fragmentation spectrum of singly protonated CycA generated by electrospray ionization (**A**) and MALDI (**B**) in FTICR mass spectrometer.

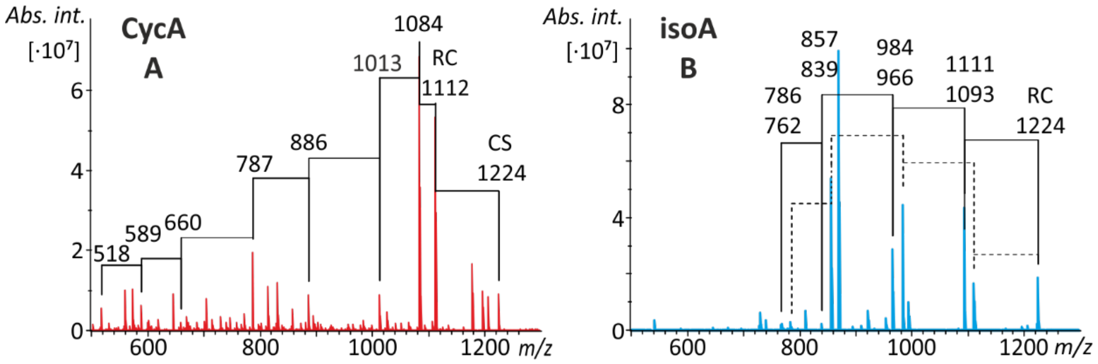

Figure S10 High mass resolution fragmentation spectra of sodiated molecules (*m/z* 1224.80) of CycA (**A**) and isoA (**B**) recorded by FTICR mass spectrometry. In the mass spectrum, peaks are displayed with their nominal masses. Exact mass measurements are provided in the table below. SC = side chain of MeBmt, RC = ring cleavage, CO = carbon and oxide.

Panel	Nominal mass	Ion name	Measured [*m/z*]	Calculated [*m/z*]
A	1013	2–3_B10-(SC+CO)	1013.7094	1013.7097
	886	2–3_B9-(SC+CO)	886.6096	886.6100
	787	2–3_B8-(SC+CO)	787.5413	787.5413
	660	2–3_B7-(SC+CO)	660.4422	660.4419
	589	2–3_B6-(SC+CO)	589.4045	589.4048
	518	2–3_B5-(SC+CO)	518.3675	518.3677
B	1111	1–11_B10	1111.7462	1111.7465
	1093	1–11_B10-H_2O	1093.7354	1093.736
	984	1–11_B9	984.6464	984.6468
	966	1–11_B9-H_2O	966.6359	966.6362
	857	1–11_B8	857.5467	857.5471
	839	1–11_B8-H_2O	839.5362	839.5365
	786	1–11_B7	786.5097	786.5010
	768	1–11_B7-H_2O	768.4991	768.4994

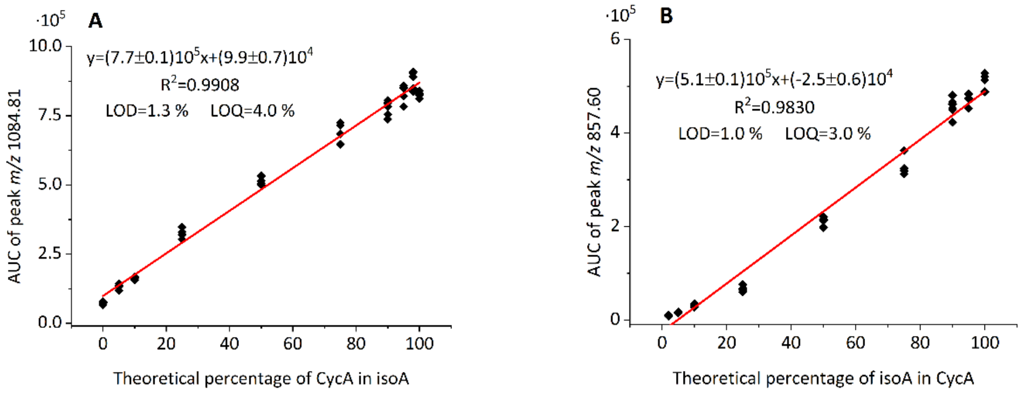

Figure S11 Calibration curves for CycA in isoA (A) and isoA in CycA (B). The plots were constructed by calculating the average area under the curve (AUC) in mobilograms of their characteristic fragments at *m/z* 1084.81 (CycA) and 857.60 (isoA). ± refers to the standard deviation.

Table S1 The ^{1}H, ^{13}C, and ^{15}N NMR data of the main conformational isomer of isoA (TEVA standard). Data were collected on NMR spectrometer - Bruker Avance III 600 MHz (600.23 MHz for ^{1}H, 150.94 MHz for ^{13}C, 60.82 MHz for ^{15}N, CD_2Cl_2, 293.2 K).

Atom	δ_C	δ_N	δ_H	n_H	m.	J_{H-H} [Hz]	diagnostic HMBC (C to H or N to H)
**1 α**	66.44	-	3.143	1	d	7.2	1 N-CH_3
**1 β**	79.34	-	4.937	1	dd	7.2, 8.8	-
**1 γ**	35.45	-	1.891	1	m	-	-

**1 δ**	34.60	-	2.337	1	m	-	-
			1.769	1	m	-	
**1 ε**	129.59	-	5.38 [H]	1	m	-	-
**1 ζ**	127.10	-	5.43 [H]	1	m	-	-
**1 η**	18.10	-	1.642	3	d	6.0	-
**1 γ - CH_3**	16.86	-	0.88 [H]	3	m	-	1β, 1γ, 1δ
**1 NH**	-	23.9	n.d.	-	-	-	1α, 1β, 1 N-CH_3
**1 N-CH_3**	35.16	-	2.275	3	s	-	1α
**1 CO**	171.53	-	-	-	-	-	1α, 2NH, 1β[w]
**2 α**	49.96	-	4.902	1	ddd	8.9, 7.6, 5.7	-
**2 β**	26.29	-	1.81 [H]	1	m	-	2α
			1.59 [H]	1	m	-	
**2 γ**	10.19	-	0.92 [H]	3	m	-	2α
**2 NH**	-	116.0	7.758	1	d	8.9	2α, 2β
**2 CO**	172.93	-	-	-	-	-	2α, 3α, 3 N-CH_3, 2-NH[w]
**3 α**	50.18	-	4.806	1	d	16.4	3 N-CH_3
			3.539	1	d	16.4	
**3 N**	-	103.0	-	-	-	-	3α, 3 N-CH_3
**3 N-CH_3**	36.93	-	3.085	3	s	-	3α
**3 CO**	168.54	-	-	-	-	-	3α, 4α, 4 N-CH_3
**4 α**	55.72	-	5.132	1	dd	10.1, 5.3	4β, 4 N-CH_3
**4 β**	36.48	-	1.80 [H]	1	m	-	-
			1.57 [H]	1	m	-	
**4 γ**	25.34	-	1.40 [H]	1	m	-	-
**4 δ1**	21.83	-	0.877	3	d	6.6	-
**4 δ2**	23.38	-	0.93 [H]	3	m	-	-
**4 N**	-	109.4	-	-	-	-	4α, 4β, 4 N-CH_3
**4 N-CH_3**	30.39	-	2.863	3	s	-	4α
**4 CO**	170.59	-	-	-	-	-	4α, 5α, 5-NH, 4β[w]
**5 α**	54.31	-	4.728	1	dd	9.0, 7.7	5-NH
**5 β**	31.39	-	2.032	1	m	-	-
**5 γ1**	19.82	-	0.90 [H]	3	m	-	-
**5 γ2**	18.22	-	0.88 [H]	3	m	-	-
**5 NH**	-	115.6	7.139	1	d	9.0	5α, 5β
**5 CO**	173.45	-	-	-	-	-	5α, 6 N-CH_3, 5-NH[w], 6α[w]
**6 α**	54.92	-	5.073	1	dd	8.6, 6.6	6 N-CH_3
**6 β**	36.00	-	1.68 [H]	1	m	-	-
			1.60 [H]	1	m	-	
**6 γ**	25.06	-	1.44 [H]	1	m	-	-
**6 δ1**	22.24	-	0.840	3	d	6.5	-

**6 δ2**	23.21	-	0.91 [H]	3	m		-
**6 N**	-	115.9	-	-	-	-	6α, 6β, 6 N-CH_3
**6 N-CH3**	31.19	-	2.985	3	s	-	6α
**6 CO**	170.31	-	-	-	-	-	6α, 6β, 7-NH, 7α[w]
**7 α**	48.92	-	4.427	1	dq	7.8, 7.1	7β, 7-NH
**7 β**	18.42	-	1.241	3	d	7.1	7α
**7 NH**	-	120.4	6.676	1	d	7.8	7α, 7β
**7 CO**	171.64	-	-	-	-	-	7α, 7β, 8α, 8-NH
**8 α**	45.70	-	4.787	1	dq	8.2, 6.9	8β, 8-NH
**8 β**	17.74	-	1.273	3	d	6.9	8α, 8-NH
**8 NH**	-	119.2	6.741	1	d	8.2	8α, 8β
**8 CO**	172.86	-	-	-	-	-	8α, 8β, (9α), 9 N-CH_3, 8-NH[w]
**9 α**	52.09	-	5.45 [H]	1	m	-	9β, 9γ, 9 N-CH_3
**9 β**	38.07	-	1.58 [H]	2	m	-	-
**9 γ**	25.06	-	1.37 [H]	1	m	-	9β
**9 δ1**	22.35	-	0.90 [H]	3	m	-	9β
**9 δ2**	23.41	-	0.92 [H]	3	m	-	9β
**9 N**	-	110.9	-	-	-	-	(9α), 9β, 9 N-CH_3
**9 N-CH_3**	30.31	-	2.934	3	s	-	(9α)
**9 CO**	171.47	-	-	-	-	-	(9α), 9β, (10α), 10 N-CH_3
**10 α**	51.52	-	5.45 [H]	1	m	-	10β, 10γ, 10 N-CH_3
**10 β**	37.88	-	1.71 [H]	1	m	-	-
			1.45 [H]	1	m	-	
**10 γ**	25.20	-	1.469	1	m	-	10β
**10 δ1**	21.83	-	0.914	3	m	-	10β
**10 δ2**	23.56	-	0.992	3	d	6.2	10β
**10 N**	-	114.7	-	-	-	-	(9α), (10α), 10β, 10 N-CH_3
**10 N-CH_3**	31.02	-	2.946	3	s	-	(10α)
**10 CO**	173.04	-	-	-	-	-	(10α), 10βd 11α, 11 N-CH_3, 10βu[w]
**11 α**	64.31	-	4.326	1	d	10.0	11β
**11 β**	27.63	-	2.266	1	dqq	10.0, 6.8, 6.6	11α
**11 γ1**	19.49	-	0.860	3	d	6.8	11α, 11γ2
**11 γ2**	20.49	-	1.042	3	d	6.6	11α, 11γ1
**11 N**	-	112.8	-	-	-	-	11α, 11β, 11 N-CH_3
**11 N-CH_3**	32.96	-	3.090	3	s	-	11α
**11 CO**	171.29	-	-	-	-	-	11α, 11β, 1β

[H]: HSQC readout, [w]: weak correlation, n.d.: not detected, (): ambiguous correlation due to proton signal overlap
